# Transmitter and receiver of the low frequency horseshoe bat *Rhinolophus paradoxolophus* are functionally matched for fluttering target detection

**DOI:** 10.1007/s00359-022-01571-0

**Published:** 2022-09-22

**Authors:** Diana Schoeppler, Katrin Kost, Hans-Ulrich Schnitzler, Annette Denzinger

**Affiliations:** Animal Physiology, Institute for Neurobiology, Faculty of Science, University of Tübingen, Tübingen, Germany

**Keywords:** Echolocation, Doppler shift compensation, Auditory fovea, Behavioral audiogram

## Abstract

Flutter-detecting foragers require specific adaptations of the transmitter and the receiver of their echolocation systems to detect and evaluate flutter information in the echoes of potential prey. These adaptations include Doppler shift compensation (DSC), which keeps the echo frequency from targets ahead constant at a reference frequency (f_ref_), and an auditory fovea in the cochlea, which results in foveal areas in the hearing system with many sharply tuned neurons with best frequencies near f_ref_. So far, this functional match has been verified only for a very few key species, but is postulated for all flutter-detecting foragers. In this study we determined both, the transmitter and receiver properties within individuals of the Bourret’s horseshoe bat (*Rhinolophus paradoxolophus*), an allometric outlier in the rhinolophid family. Here we show that the transmitter and receiver are functionally matched in a similar way as postulated for all flutter-detecting foragers. The performance of DSC, measured as the ability to keep the echo frequency constant at f_ref_, had a precision similar to that found in other flutter-detecting foragers, and the audiogram showed the characteristic course with a minimum at f_ref_. Furthermore, we show for a rhinolophid bat a variation over time of the coupled resting frequency and f_ref_. Finally, we discuss the tight match between transmitter and receiver properties, which is guaranteed by the link between the foveal areas of the receiver and the audio–vocal control system for DSC.

**Supplementary Information:**

The online version contains supplementary material available at 10.1007/s00359-022-01571-0.

## Introduction

Echolocation and powered flight enable bats to nocturnally exploit a high diversity of diets in myriad habitats. Echolocation and motor systems in bats have been adapted to where they forage and to how they find and acquire prey, with the strongest constraints on the echolocation and motor systems determined by the distances between bat, prey, and background (Schnitzler and Kalko 1998, 2001; Schnitzler et al. 2003; Denzinger and Schnitzler 2013). Bats that search for prey close to vegetation in narrow space are faced with an acoustic scene, where prey and background echoes overlap. To find and acquire prey, bats have to distinguish prey echoes among many clutter echoes, which primarily becomes a pattern recognition task (Denzinger et al. 2018). Narrow space flutter-detecting foragers discriminate between clutter and prey echoes by assessing echoes received from fluttering prey, with amplitude and frequency modulations caused by the wing movements. All members of the Rhinolophidae and Hipposideridae families, and the more phylogenetically distant mormoopid *Pteronotus parnellii*, belong to the guild of narrow space flutter-detecting foragers (Denzinger and Schnitzler 2013), a functional group containing about 200 species (Wilson and Mittermeier 2019).

It is assumed that the transmitter and receiver of all flutter-detecting foragers are functionally matched to evaluate flutter information in echoes of fluttering prey (for reviews, see Schnitzler and Ostwald 1983; Schnitzler and Denzinger 2011). However, this match has been proven so far for a very few key species. A characteristic property of the transmitter in flutter-detecting foragers is the use of multi-harmonic echolocation signals emitted at a high duty cycle, which consist of a long constant-frequency (CF) component followed by a downward frequency-modulated (FM) terminal component, with the second harmonic containing the main energy (CF_2_). Due to the high duty cycle, these flutter-detecting foragers have also been classified as high duty cycle bats (Fenton et al. 2012). Another characteristic transmitter property is the Doppler shift compensation (DSC) (Rhinolophidae: Schnitzler 1968; *P. parnellii*: Schnitzler 1970; Hipposideridae: Gustafson and Schnitzler 1979). Controlled by an audio–vocal feedback system, these bats lower the emission frequency to keep the CF_2_ of echoes from targets ahead constant (f_echo_) at a reference frequency (f_ref_, defined as the averaged f_echo_), thereby compensating the Doppler shifts (DS) induced by their own flight movement (Schnitzler 1973). Small standard deviations from f_ref_ of about 0.1–0.2% have been measured (Schnitzler and Denzinger 2011). The value under control (f_echo_) is maintained at the reference value (f_ref_) over a wide range of flight speeds, indicating a high quality of the DSC system (Schnitzler 1973; Schnitzler and Denzinger 2011; Schoeppler et al. 2018). The f_ref_ is about 150–200 Hz above the CF_2_ of stationary bats before take-off, the so-called resting frequency (f_rest_) (reviewed in Schnitzler and Denzinger 2011). This difference is termed offset (Schuller et al. 1974). Although f_ref_ and f_rest_ can change over time, e.g., owing to different body temperatures, it is a common feature of the transmitter system that f_rest_ and f_ref_ are coupled (Huffman and Henson 1993; Schoeppler et al. 2018; Zhang et al. 2019).

If the long CF call components strike a fluttering insect, the returning echoes contain short and strong amplitude and frequency modulation peaks, so called glints, that arise when the reflecting wing is perpendicular to the impinging sound waves (Schnitzler 1978; Schnitzler et al. 1983). This flutter information enables bats to distinguish prey echoes from unmodulated background echoes (Schnitzler and Henson 1980).

The receiver, which corresponds to the auditory system of flutter-detecting foragers, is especially adapted to process flutter information in echoes from prey. The auditory fovea, a specialized area of the basilar membrane in the cochlea with an expanded frequency representation around f_ref_ (*R. ferrumequinum*: Bruns 1976; Bruns and Schmieszek 1980; *R. rouxii*: Vater et al. 1985; *P. parnellii*: Kössl and Vater 1985; *Hipposideros lankadiva*: citated in Neuweiler 1990), leads to afferent projections in higher foveal areas, with an overrepresentation of sharply tuned neurons near f_ref_ (*R. ferrumequinum*: Schuller and Pollak 1979; *Hipposideros speoris*: Rübsamen et al. 1988; *P parnellii*: Pollak and Bodenhammer 1981). These neurons have the function to decode the flutter information in insect echoes and to deliver relevant feedback information to the audio–vocal control system, which determines the frequency of the emitted signals (for reviews, see Neuweiler et al. 1980; Schnitzler and Ostwald 1983; Metzner 1989; Schnitzler and Denzinger 2011).

The foveal organization of the auditory system of flutter-detecting foragers is also reflected in the behavioural and neurophysiological audiograms of these species. All audiograms measured to date exhibit a distinct threshold minimum near the f_ref_, which mirrors the high number of neurons in the foveal areas tuned to f_ref_ (Suga and Jen 1977; Ostwald 1984; for reviews see Neuweiler et al. 1980; Schnitzler and Ostwald 1983; Schnitzler and Denzinger 2011). Therefore, audiograms can serve as reliable indicators of the foveal organisation of the hearing system. In addition, all audiograms show a well-defined threshold maximum below the f_ref_ which covers the range of the emission frequencies of DSC bats.

The tight match between transmitter and receiver properties is an indispensable prerequisite for the evaluation of flutter information; however, it is postulated for all flutter-detecting foragers based on studies from very few flutter-detecting foragers. The direct match of transmitter and receiver properties has only been reported for *R. ferrumequinum* (Long and Schnitzler 1975; Schnitzler et al. 1976), and can be assumed for *R. ferrumequinum nippon* (Taniguchi 1985); *R. rouxii* (Henson et al. 1985) and the mormoopid *P. parnellii* (Henson et al. 1980, 1985), where the individual measured f_rest_ was slightly below the audiogram minimum. For other species, some details on either transmitter or receiver properties have been published, without assessment of the whole transmitter–receiver system, e.g., in *Rhinolophus philippinensis,* foveal neurons are tuned to 30–33 kHz, with Q _10 dB_ values of up to 65 (Jen and Suthers 1982).

To test the generalizability of the hypothesis that transmitter and receiver properties of flutter detection foragers are coupled, we chose an outlier horseshoe bat, *Rhinolophus paradoxolophus* that emits echolocation signals with a CF_2_ distinctly lower than predicted by allometry (Thong 2011). *R. paradoxolophus* is a medium sized species with a body mass ~ 9.5 g, a mean forearm length of ~ 51 mm and a low f_rest_ of ~ 28.5 kHz. We assume that *R. paradoxolophus* use their low frequency echolocation signals in a similar way as other flutter-detecting foragers to forage for prey in narrow space. Furthermore, we expect that the transmitter and receiver properties of *R. paradoxolophus* should be matched in a similar way as in other rhinolophids. We examined the transmitter properties as performance of DSC and the coupling of f_rest_ to f_ref_ and its variation over time. The receiver properties were determined by measuring the behavioural audiogram of *R. paradoxolophus*. Therefore, we identified the lowest amplitude over the frequency range of 15 to 100 kHz which just elicited a pinna reflex, also known as Preyer reflex. We hypothesized that—independent of the low frequency range—the course of the auditory threshold curve should be similar to that of other rhinolophids. Finally, we hypothesized that f_ref_, as determined by DSC, should predict a minimum in the behavioural audiogram at f_ref,_ thus confirming the close match between transmitter and receiver for flutter detection even in the low frequency outlier *R. paradoxolophus*.

## Materials and methods

### Animals and husbandry

Three adult, male *Rhinolophus paradoxolophus* (Bourret 1951) were captured in Northern Vietnam (permission No. 192/STTNSV from May 13th 2009 granted to the Vietnamese Institute of Ecology and Biological Resources, Hanoi). At the University of Tübingen (Germany) the bats were housed in a tent (2.3 × 1.4 × 1.25 m) under a constant temperature of 26 ± 2 °C, a constant humidity of 60 ± 5%, and a light:dark cycle of 12:12 h. Bats were fed with mealworms (*Tenebrio molitor* larvae), supplemented with minerals (Korvimin®, WDT eG, Germany), vitamins (Nutri-Cal® Albrecht GmbH, Germany) and fatty acids (Efaderm® aristavet GmbH & Co., Germany). Water was offered ad libitum. All experiments were conducted at the beginning of the dark phase of the daily cycle.

### Ethical statement

All experiments were performed in accordance with the German Animal Welfare Legislation. The license to keep *Rhinolophus paradoxolophus* was issued by the responsible agency (Regierungspräsidium Tübingen, Germany).

### Measurement of doppler shift compensation

#### Training procedure and data acquisition

Experiments were conducted in a flight room (6 × 3.5 × 3 m). Bats were rewarded with mealworms for correct behaviour. Training sessions lasted between 30 and 45 min, and ended when the bat was satiated, usually after ~ 10 mealworms. After the training sessions bats had ad libitum access to food in the housing tent for the rest of the dark phase to make sure that every bat could fully supply itself with food. At days without training, bats had ad libitum access to food. The weight of the bats was daily monitored.

Bats were trained to fly from a starting bar positioned 1.5 m above the floor to a landing grid attached to the ceiling at a height of 3 m. The direct distance between start and landing was 3.7 m. Echolocation and flight behaviour were recorded with a synchronized video and sound recording system (PC-Tape system, University of Tübingen, Germany). The echolocation signals were picked up with a custom-made ultrasonic microphone (University of Tübingen, Germany; flat frequency response: 15–75 kHz ± 2 dB SPL), which was positioned behind the landing grid. Signals were amplified by 20 dB and stored as.wav files (480 kHz, 16 bit). The flight behaviour was recorded with two infrared cameras (Sanyo IR CCD, Panasonic, Osaka, Japan, rate 50 Hz). Each half-frame was illuminated for 1 ms with two infrared strobe flashes (University of Tübingen, Germany) positioned on the cameras. The video data were stored on tapes using camcorders (Sony, DCR-TR V50E, Tokio, Japan).

### Data analysis

From video recordings, we reconstructed the three-dimensional flight path (SIMI® Motion Reality Motion Systems, 7.5.293, Germany) and calculated the flight speed of bats (reconstruction error of 1.0 cm on average and 2.8 cm at highest). Sound analysis was conducted with the custom-written software Selena (Animal Physiology, University of Tübingen, Germany). Signals were displayed as colour sonagrams (FFT 512, Blackman, dynamic range 90 dB) in a window of 512 × 512 pixels with a frequency range of 0–50 kHz and a duration of 120 ms. Due to interpolation in time and auto padding, we reached a resolution of *Δ*f = 97.9 Hz and *Δ*t = 0.23 ms. With a custom Matlab® routine (MathWorks® USA, 2013b) written by Peter Stilz (University of Tübingen) we analysed the signal parameters (signal duration and pulse interval) and define the beginning and end of the signal at 30 dB below the peak amplitude. Pulse interval was defined as the time from the beginning of one signal to the beginning of the prior signal, and duty cycle as the percentage of time filled with signal. For precise CF_2_ measurements, which were necessary to determine the DSC, signals were analysed with an FFT of 8192 points (zero padding), displayed in the frequency range of 25–31 kHz and a duration of 200 ms, which resulted in a frequency resolution of 11.5 Hz. CF_2_ was measured at the peak amplitude of the CF component.

The resting frequency (f_rest_) was measured prior to each flight, as averaged CF_2_ of the last 20 calls before take-off. The CF_2_ of the emitted signals (f_s_) was calculated from the microphone frequency (f_m_ = frequency recorded at the microphone) by evaluating the Doppler shift (DS) produced by the approaching bat according to its flight speed (v_b_) and the velocity of sound (*c* = 343 m/s) using Eq. (1) published by Schnitzler (1973):

1 $$\mathrm{fs }=\mathrm{ fm}\times \frac{\left(\mathrm{c}-\mathrm{vb}\right)}{\mathrm{c}}$$

In situations, where bats did not fly straight toward the microphone, the DS was lower than predicted from the bat’s flight speed. Therefore, we measured the angle α between flight and microphone direction and used the cosine of α to correct the DS and to calculate the emission frequency with

2 $$\mathrm{femission}=\mathrm{fs}-\mathrm{DS}\times \mathrm{cos}\left(\mathrm{\alpha }\right)$$

The echo frequency (f_echo_) from stationary targets was calculated with Eq. (3) by adding two DS (Schnitzler 1973):

3 $$\mathrm{fecho }=\mathrm{ femission}\times \frac{(\mathrm{c}+\mathrm{vb})}{(\mathrm{c}-\mathrm{vb})}$$

The reference frequency (f_ref_) was determined as the averaged echo frequency of each flight, and the difference between f_ref_ and f_rest_ is defined as the offset.

## Statistical analysis

Statistical analysis was performed in JMP® (SAS Institute GmbH, Heidelberg, Germany). For each bat we analysed 10 flights. We compared the signal parameters: signal duration, pulse interval and duty cycle of orientation flight, where single signals were produced, and terminal group, between individuals. We controlled for normal distribution using normal quantile plots, if significant, nonparametric tests were calculated. The nonparametric signal parameters were tested with Kruskal–Wallis tests to determine if there were differences between individual bats. Furthermore, we tested the coupling of f_rest_ and f_ref_ in each bat with a Pearson correlation. We measured the precision of DSC by calculating the deviation of the f_echo_ of each call from the f_ref_ of the corresponding flight. We displayed this deviation in relation to the flight speed. We calculated means of deviations within flight speed classes of 0.25 m/s for each bat, i.e., 15, 13, and 14 classes for bats 1, 2, and 3, respectively. These means of the three bats between flight speed classes were compared with an ANOVA and Tukey–Kramer test.

### Measurement of the behavioural audiogram

#### Training procedure and data acquisition

The behavioural audiogram was measured by determining the threshold SPL which just elicited a Preyer reflex at different frequencies. The Preyer reflex consists of a characteristic ear twitch in response to a weak auditory stimulus and can only be measured in a calm and relaxed animal. The study was carried out with one bat (bat 1). Measurements were conducted in a recording box (60 × 45 × 36 cm) in the flight room. Bats were familiar with the box, as it was initially used to train the bats to feed on mealworms and used as temporary housing during cleaning. For measurements, the box was covered with foam and a soft grid (12.5 × 12.5 cm) was attached to the ceiling to offer a preferred resting site. In the box, bats had free access to water, and food was regularly offered. The audio and video systems were positioned at one side of the box in alignment to the bat’s position on the resting grid.

Prior to transferring the bat into the recording box, it was allowed to fly for 1 to 2 h together with conspecifics in the flight room. In the recording box, individuals calmed down within a few minutes. Stimulus presentation started when the bat rested calmly at the grid without moving its ears and without emitting echolocation signals. Sessions ended when the bat started to demonstrate increased activity within the box.

#### Stimulus generation and video recordings

For stimulus generation we used a custom Matlab® routine (MathWorks® USA, R2011b) written by Manfred Kössl (University of Frankfurt). The pure tone stimulus was generated with a sound card (Quartet® Infrasonic, multi-purpose 4 × 4 channel 24 bit/192 kHz, Serial No. SMC080501737; Newegg, CA, USA), amplified (Krohn-Hite®, Model 7500, MA, USA) and played back through a custom-made loudspeaker (University of Tübingen, Germany). The loudspeaker was calibrated (Audio Wave Analyzer, Rhode & Schwarz, 1/8 inch Brüel & Kjær® microphone, Nærum, Denmark) and had a flat frequency response from 15 to 110 ± 1.5 kHz up to 95 dB rms SPL re 40 cm in front of the speaker, which corresponded to the distance between loudspeaker and tested bat in the setup.

Stimuli always had a duration of 30 ms with a slope of 1.5 ms. Thresholds were measured at frequencies from 15 to 95 kHz in 5 kHz steps except around the second and fourth harmonic of the echolocation signals (CF_2_ and CF_4_), where smaller steps were used (measured frequencies: 24.3 kHz, 26.3 kHz, 28.3 kHz, 28.55 kHz, 28.8 kHz, 29.05 kHz, 29.3 kHz, 29.8 kHz, 30.3 kHz, 31.3 kHz, 32.3 kHz, 36 kHz, 58.1 kHz, 58.6 kHz, 59.1 kHz, 62.6 kHz, 63.6 kHz, 64.6 kHz and 67.4 kHz). In total, the threshold was determined for 35 frequencies. Except around CF_2_, each frequency threshold was measured with an ascending staircase procedure at least three times, starting with stimulus amplitude of 30 dB SPL. The low amplitude ensured that no startle response occurred, even at frequencies with lower thresholds. Amplitude was increased in 5 dB SPL steps until the pinna reflex was elicited. If the bat already responded at 30 dB SPL, we reduced the amplitude to sub-threshold level. Around the CF_2_ we started with stimulus amplitude of 5 dB SPL and used 1 dB SPL steps. Similarly, around the CF_4_ we also measured in 1 dB SPL steps. To avoid habituation, we used the highest possible difference for consecutive stimulus frequencies, and stimulus intervals of at least 2 min.

### Data analysis

At the beginning of each session, resting signals were recorded for 5–10 s. Resting signals, stimulus and the behaviour of the bat were recorded with the PC-tape system and the synchronized infrared video system as described above but with only one camera equipped with a zoom objective (Computar TV Zoom Lens, H16Z7516M, 7.5–120 mm; NC, USA). Video tapes were digitized and analysed in SIMI° Motion (SIMI® Motion Reality Motion Systems, 7.5.293, Germany). For analysis of the pinna reflex, the distance between a fixed point on the head between the two ears and the tip of the respective ear was measured at each half-frame (*Δ*t = 0.02 ms and *Δ*d = 1.2 mm). A positive reaction of the bat was defined as a pinna movement of at least 0.01 in the coordinates of the SIMI program, which corresponded to 1.2 mm, calibrated with ear length (Fig. S1). Pinna movement was analysed from 0.2 s before to 1 s after the stimulus. Each frequency of the audiogram was measured up to five times and displayed as a mean with standard error (± SEM).

## Results

### Echolocation behaviour

**Fig. 1:**
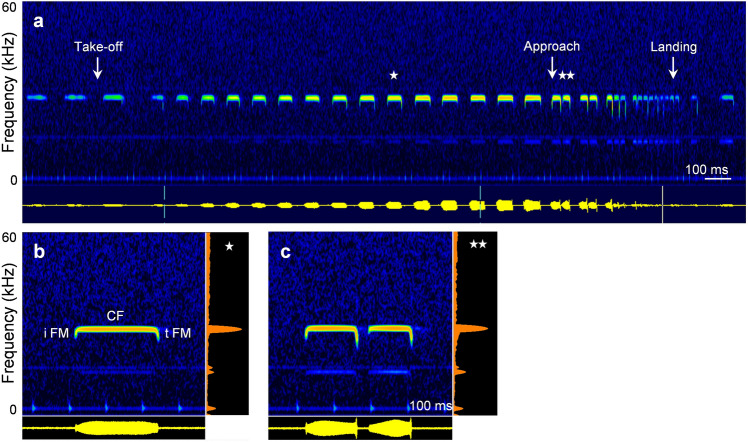
Sonagram and oscillogram of an echolocation sequence of bat 1 flying to a landing grid (**a**) and representative signals with averaged power spectra (**b**, **c**). Signals of orientation flight (**b**) and approach flight (**c**) are taken from a (marked with asterisks). iFM = initial frequency-modulated component, CF = constant-frequency component and tFM = terminal frequency-modulated component

All *R. paradoxolophus* flew on a typical path from the starting bar to the landing grid. During orientation flight, from start to the begin of the approach, bats emitted long signals, with the main energy in the second harmonic (Fig. 1a). Signals started with a short initial FM, followed by a long CF and a short terminal FM (Fig. 1b). The calls had an average duration of 45–50 ms, pulse interval of 86–87 ms and duty cycle of 49–53% (Table 1). All signal parameters during orientation flight differed significantly between individual bats (Kruskal–Wallis *χ*^2^_(2)_ ≥ 9.7; *p* ≤ 0.0079).

**Table 1:** Echolocation signal parameters during orientation flight and in the terminal group of the approach flight (mean ± s.d. (*N*) and *p*-values (Kruskal–Wallis))

	Bat	Orientation flight	Terminal group
Signal duration (ms)	1 2 3	46.3 ± 6.4 (188) 45.0 ± 8.5 (168) 50.2 ± 11.4 (145)	12.3 ± 1.5 (94) 12.0 ± 2.0 (103) 12.4 ± 1.9 (68)
		*p* < 0.0001	*p* = 0.1626
Pulse interval (ms)	1 2 3	86.4 ± 9.5 (178) 85.9 ± 13.4 (158) 87.2 ± 20.2 (135)	18.9 ± 8.5 (94) 16.5 ± 7.1 (103) 20.1 ± 7.7 (67)
		*p* = 0.0079	*p* < 0.0001
Duty cycle (%)	1 2 3	50.4 ± 13.6 (188) 49.1 ± 15.0 (168) 52.7 ± 18.9 (145)	76.0 ± 5.4 (84) 84.4 ± 10.6 (93) 71.7 ± 7.4 (58)
		*p* < 0.0001	*p* < 0.0001

During approach, signals were arranged in groups. The approach started with a group of two signals (Fig. 1a, c) and ended with a long terminal group of seven to 10 signals on average and a maximum of 13 signals. With the beginning of the approach, signal duration and pulse interval decreased to values of 12 ms and 17–20 ms, respectively, and duty cycle increased to 72–84% in the terminal group (Table 1). While the signal duration did not differ (Kruskal–Wallis *χ*^2^_(2)_ = 3.6; *p* = 0.16), the pulse duration and duty cycle differed between individuals (Kruskal–Wallis *χ*^2^_(2)_ ≥ 67.1; *p* < 0.0001).

### Doppler shift compensation system and frequency variation

*R. paradoxolophus* emitted echolocation calls with a rather low species-specific CF_2_, which is typical for members of the *philippinensis* group. The average individual resting frequencies (f_rest_) within a recording period of 3 weeks were 28.5 kHz (bat 1), 28.35 kHz (bat 2) and 28.44 kHz (bat 3). Between days, the mean f_rest_ varied over the whole recording time by up to 60–90 Hz. However, the standard deviations of the 20 signals before each flight were small, and measured between 20 Hz (bat 2) or 30 Hz on average (bat 1 and bat 3), with a maximum of 40 Hz.

**Fig. 2:**
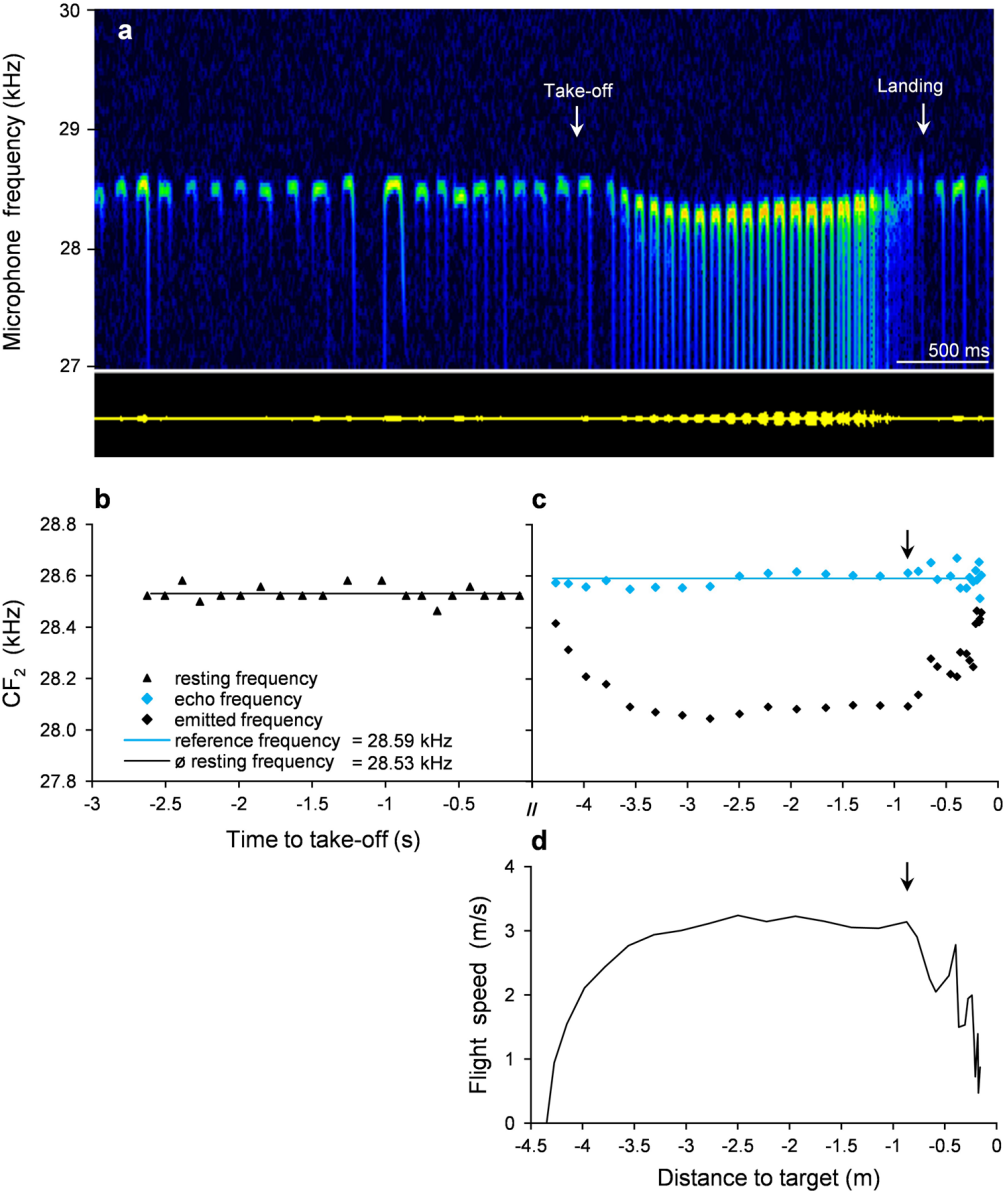
Doppler shift compensation of the representative flight shown in Fig. 1a. **a** Sonagram with a high frequency resolution (FFT 16,384) demonstrating the resting frequency before take-off and the lowering of the microphone frequency during flight. **b** CF_2_ of the last 20 echolocation signals before take-off (f_rest_), (**c**) emitted frequency and echo frequency during flight, calculated for targets ahead using the flight speed (**d**). The reference frequency corresponds to the averaged echo frequency. The offset measured 60 Hz. Arrows indicate the beginning of the approach phase

**Fig. 3:**
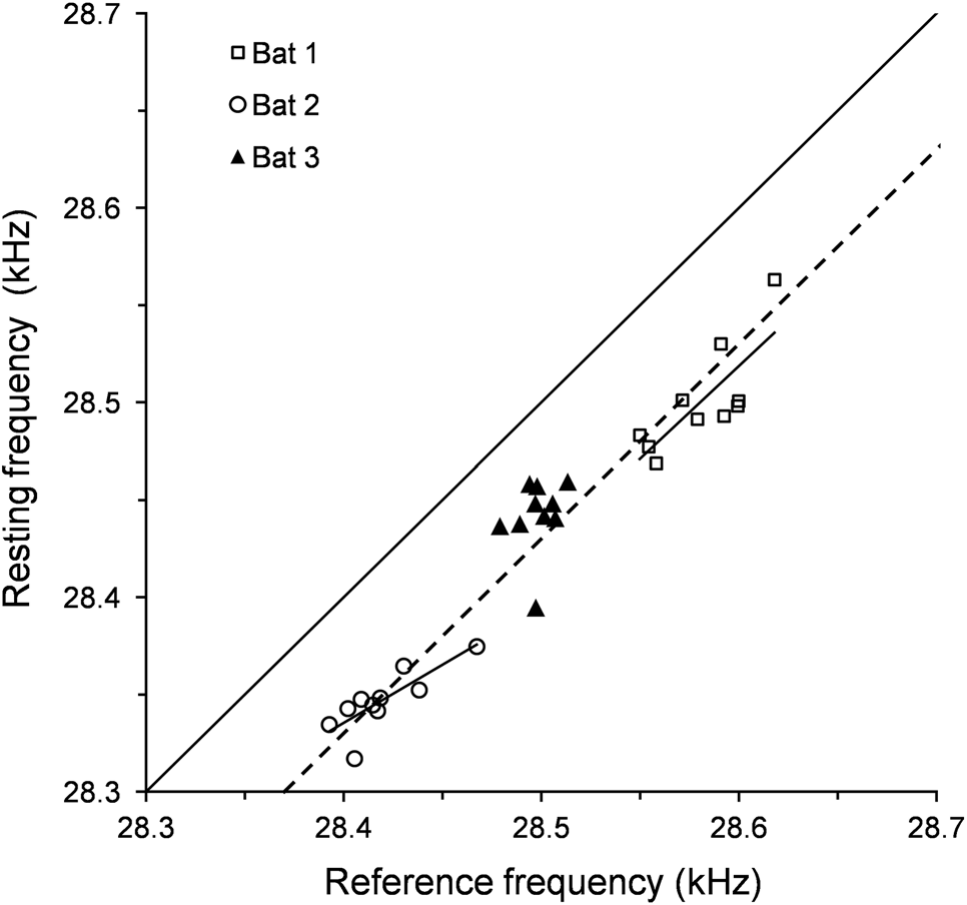
Coupling between the means of the resting and reference frequencies, with variations over 3 weeks. For each bat, 10 flights with linear regression lines are shown. The solid line indicates the angle bisector, and the dotted line indicates the average offset of 70 Hz

In flight to a stationary target, *R. paradoxolophus* lowered the emission frequency in such a way that the calculated frequency of the CF_2_ of the echoes from ahead (f_echo_) was maintained constant at a f_ref_ (Fig. 2). The averaged standard deviation of f_echo_ was 60 Hz (bat 1: 70 Hz, bat 2: 60 Hz, bat 3: 50 Hz), and ranged from 40 to 120 Hz. The offset between the f_rest_ and the f_ref_ was 70 Hz (bat 1: 80 Hz, bat 2: 70 Hz, bat 3: 60 Hz), which corresponds to 0.25% of CF_2_. A variation of f_ref_ of 70–80 Hz at maximum in bat 1 and 2 and 30 Hz in bat 3 was observed between trial days.

f_rest_ and f_ref_ varied over time but were tightly coupled, which is indicated by regression lines running almost parallel to the bisector (*r*^2^(8) ≥ 0.62, *p* ≤ 0.0067) in the correlations of f_rest_ and f_ref_ of bat 1 and bat 2 (Fig. 3). With a maximum variation of 20 Hz and 30 Hz the f_rest_ and f_echo_ of bat 3 varied less (omitting a single outlier of almost 70 Hz).

**Fig. 4:**
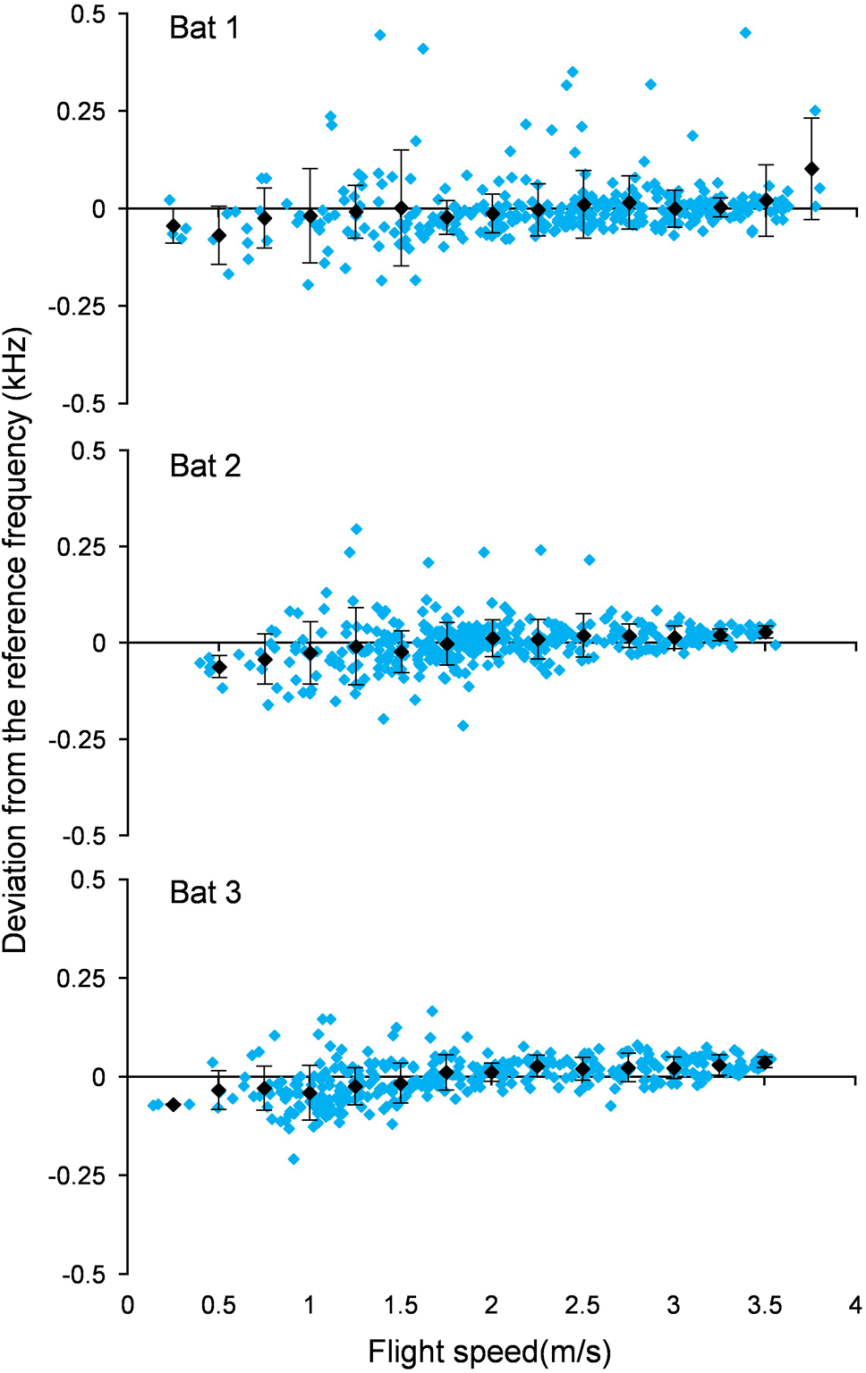
Precision of Doppler shift compensation behaviour in *R. paradoxolophus*. Deviation of the echo frequency of each call from the reference frequency of the corresponding flight was calculated and plotted against the flight speed. Black diamonds indicate means (± s.d.) calculated for 0.25 m/s classes. Number of flights per bat = 10

The precision of the DSC feedback control system of a species is described by the accuracy with which f_echo_ is kept at f_ref_, independent of the encountered DS and thus independent of flight speed. In *R. paradoxolophus*, f_echo_ was influenced by the flight speed [F(14,27) = 17.90, *p* < 0.0001]. However, f_echo_ showed no significant changes for moderate flight speeds between 1.75 and 3.5 m/s (Tukey–Kramer *p* < 0.05 n.s. for flight speeds between 1.75 and 3.5 m/s) (Fig. 4). At lower flight speeds (< 1.75 m/s), the means tended to be below the f_ref_ with higher standard deviations (Fig. 4).

### Behavioural audiogram

**Fig. 5:**
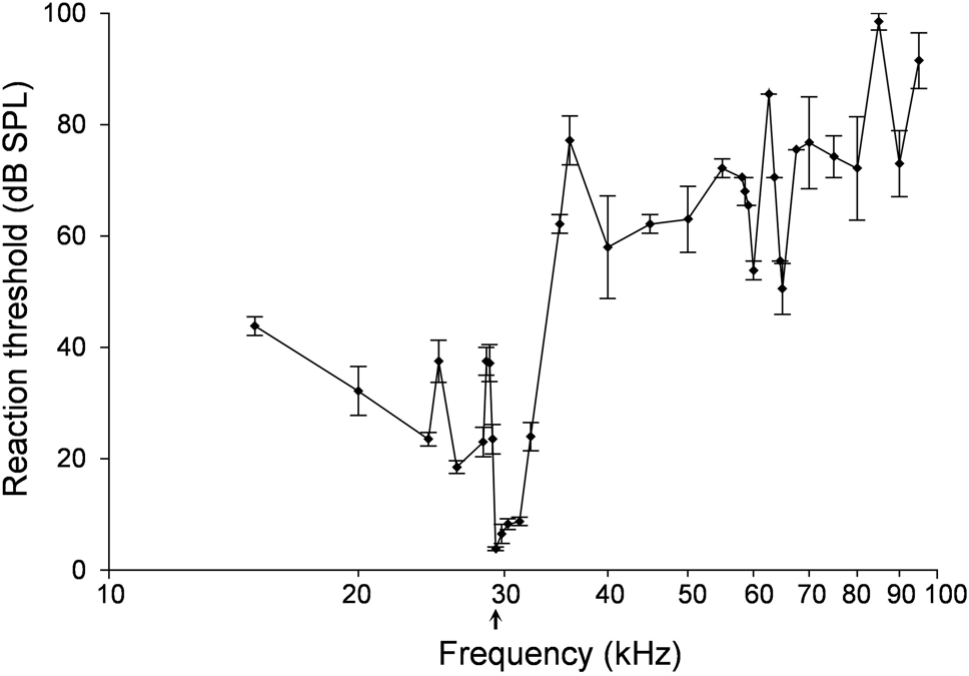
Behavioural audiogram of *Rhinolophus paradoxolophus* (bat 1) measured with the Preyer reflex (Mean ± SEM). The minimum of the audiogram at 29.3 kHz (arrow) was 140 Hz above the resting frequency of the tested individual

The audiogram of bat 1 measured with the Preyer reflex revealed a sharp minimum at 29.3 kHz with a threshold value of 3.8 ± 0.3 dB SPL (± SEM) (Fig. 5). Below the threshold minimum the threshold increased to a maximum at 28.55 to 28.88 kHz, which was approximately 35 dB SPL higher. To lower frequencies the threshold decreased again to 18.5 dB SPL at 26.3 kHz. Around the frequency range of the first harmonic, at 15 kHz, the threshold was 44 dB SPL. The threshold increase from the minimum toward higher frequencies was less steep when compared the increases toward lower frequencies. At frequencies slightly higher than the minimum frequency up to 31.3 kHz, the threshold increased gradually by 1–3 dB SPL, followed by a steep increase at frequencies from 32.3 to 36 kHz, where the maximum was reached with 77 ± 4.4 dB SPL (± SEM). At higher frequencies the threshold level stayed between 50 to 100 dB SPL. The mean f_rest_ measured in this experiment was 29.16 kHz, thus 140 Hz below the threshold minimum. While it should be noted that the behavioural audiogram was measured 12 months earlier than the DSC in the flight experiment, in the same bat the f_rest_ dropped by ~ 700 Hz from 29.16 kHz to 28.5 kHz indicating a long-term frequency variation.

## Discussion

Studies on the matching of transmitter and receiver properties of the echolocation systems of flutter-detecting foragers have, in the past, been limited to a few key species. Within the Rhinolophidae, studies have been conducted mainly on *Rhinolophus ferrumequinum*, *Rhinolophus rouxii, *and *Rhinolophus ferrumequinum nippon*. The findings of these studies were generalized to all Rhinolophids, and more broadly still to all flutter-detecting foragers. Here we present results from an outlier rhinolophid, *Rhinolophus paradoxolophus*, which differs in its allometric relationship between body size and call frequency by emitting calls with a much lower CF_2_ (Thong 2011). The aim of this study was to test the generalizability of the hypothesis that transmitter and receiver properties of the outlier *R. paradaxolophus* are matched to each other in a similar way as has been reported for other rhinolophids.

### Echolocation behaviour

A typical feature of the transmitter of flutter-detecting foragers is the emission of long calls with a constant frequency component emitted at high duty cycle (Schnitzler and Denzinger 2011). Besides the low CF_2_ of the echolocation calls, flight and echolocation behaviour of *R. paradoxolophus* corresponded to that of other rhinolophids when approaching a landing site. In orientation flight, *R. paradoxolophus* used around 50 ms-long single signals at a duty cycle of 50%, which is concordant with other rhinolophids emitting signals with durations of 27–65 ms at duty cycles of 41–57% in orientation flight, or when calling from perches (Schnitzler 1968; Tian and Schnitzler 1997; Fenton et al. 2012). The arrangement of calls in groups during approach was similar to that described for landing *R. ferrumequinum* (Schnitzler 1968; Tian and Schnitzler 1997) and the signal parameters duration, pulse interval, and duty cycle also varied in similar ways. As do other rhinolophids, *R. paradoxolophus* operates using signals with a strong second harmonic, while the first and higher harmonics are suppressed (Fig. 1). In previous studies, it was stated that the echolocation signals of *R. paradoxolophus* and *Rhinolophus rex*, another member of the *philippinensis* group, have the main energy at the first harmonic (Eger and Fenton 2003; Huihua et al. 2003; Zhang et al. 2009). Presumably, the amplitude of the first and the higher harmonics was so small in these recordings that they may not have appeared in spectrograms.

### Doppler shift compensation and frequency variation

Overall, our study confirmed a similar DSC behaviour in *R. paradoxolophus* as in other flutter-detecting foragers studied. As in other high duty cycle bats, f_ref_ was higher than f_rest_ and the two frequencies were coupled. This offset between f_rest_ and f_ref_ in *R. paradoxolophus* was comparably low (with an average of ~ 70 Hz), but measured ~ 0.25% of the f_rest_, which is similar to that of other flutter-detecting foragers (Schnitzler and Denzinger 2011) (Table 2). The ability of *R. paradoxolophus* to keep f_rest_ constant with a deviation of about 30 Hz (corresponding to 0.11% of the f_rest_) is also similar to that of other high duty cycle bats.

**Table 2:** Comparative overview of offset, standard deviations, and bandwidth

	*R. paradoxolophus*	*R. ferrumequinum*	*R. ferrumequinum nippon*^3^	*H. armiger*
f_rest_	28.4 kHz	83.0 kHz^1^		65.5 kHz^4^
Offset	0.07 kHz (0.25%)	0.15–0.20 kHz^1,2^ (0.18–0.24%)	0.14 kHz (0.2%)	0.12 kHz^3^ 0.08 kHz (0.12%)^4^
SD of f_rest_	0.03 kHz (0.11%)	0.05 kHz^1^ (0.06%)		0.14 kHz (0.21%)^4^
SD of f_**ref**_	0.06 kHz (0.21%)	0.1–0.2 kHz^2^ (0.12–0.24%)	0.11%	0.17%^3^ 0.11 kHz (0.17%)^4^

Data from *Rhinolophus paradoxolophus* (this study), *Rhinolophus ferrumequinum* (^1^Schnitzler 1968; ^2^Schnitzler and Denzinger 2011), *R. ferrumequinum nippon* (^3^Zhang et al. 2019) and the hipposiderid bat *Hipposideros armiger* (^4^Schoeppler et al. 2018; ^3^Zhang et al. 2019). The percentage values refer to the resting frequency (f_rest_) of the species

One approach taken to define the precision of DSC has been to determine the degree of undercompensation in bats sitting on a pendulum or other moving device as a percentage of the induced reduction of emission frequency in relation to the DS caused by the forward movement of the subject (Habersetzer et al. 1984; Gaioni et al. 1990; Keating et al. 1994). We think that this approach is not necessarily the best measure to assess the quality of a feedback control system. To describe the performance of DSC we propose to measure the precision with which the parameter under control (i.e., f_echo_) is kept constant at the reference parameter (i.e., f_ref_), at different flight speeds. To get comparable precision values within and between bat species, we propose to measure the precision with which f_ref_ is kept constant at different flight speeds as a percentage of the standard deviation of f_ref_ in relation to CF_2_. A similar approach to describe DSC precision was used by Zhang et al. (2019), but without referring to different flight speeds. In our subjects, the flight speed varied from almost 0 m/sec at the start and at landing to a maximum of 3.8 m/s. Playback experiments with simulated DS revealed that the feedback control system for DSC had high time constants and is, therefore, rather slow (Simmons 1974; Schuller et al. 1975). Hence, we suggest that the precision should only be measured in situations, where the real or simulated DS do not change too fast. We recommend that recordings, where fast changing DS do occur, e.g., sinusoidal changes in playback experiments, strong positive or negative accelerations in starting and landing bats, or when subjects are sitting on a pendulum, should be excluded from estimates of DSC precision. The delayed adjustment of echo CF_2_ during start and landing explains the higher standard deviations of the f_echo_ at low flight speeds found in *R. paradoxolophus* and in *Hipposideros armiger* (Schoeppler et al. 2018).

The small standard deviation of 60 Hz in f_ref._ corresponded to a precision of 0.21%, which is in the range of the values measured in other rhinolophids, in *P. parnellii* (Schnitzler and Denzinger 2011) and also in hipposiderids (Schoeppler et al. 2018; Zhang et al. 2019) (Table 2). This shows that the precision of DSC in *R. paradoxolophus* is similar to that of other flutter-detecting foragers.

In *R. paradoxolophus,* the coupled pair of f_rest_ and f_ref_ varied over time. We observed small variations (a maximum of 30 Hz) within a recording session, lasting approximately 30 min. Variation between days was higher, and measured up to 90 Hz. This variation had no impact on the offset, since f_rest_ and f_ref_ were always coupled. The variation of the coupled pair f_rest_ and f_ref_ has previously been found in *P. parnellii*, *R. ferrumequinum nippon* and in *Hipposideros armiger* (Huffman and Henson 1993; Schoeppler et al. 2018; Zhang et al. 2019) and we suggest that the coupled variation is typical for all flutter-detecting foragers. We assume that the small reversible variations in f_rest_ and f_ref_ were due to variable physiological parameters, such as body temperature, which was already described for other DS compensating bats, including *P. parnellii* and *H. armiger* (Huffmann and Henson 1993; Schoeppler et al. 2022). Body temperature most likely changes the mechanical properties of the basilar membrane, which would result in a different stimulation of the foveal area in the cochlea. This activates the audio–vocal feedback control system and leads to readjustments of the emission frequency.

Several studies have also documented individual CF_2_ variations in adult rhinolophid bats in experiments lasting several days or months (Schuller et al. 1974; Hiryu et al. 2008; Furusawa et al. 2012). Besides small reversible changes in frequency, we also observed a large non-reversible frequency drop of 700 Hz in bat 1 between the measurement of the behavioural audiogram and flight experiments, which were about 12 months apart. This bat was in good body and health condition, but the change in frequency might be caused by a morphological change of the cochlea due to the aging process, similar to the observation in old *R. ferrumequinum* in the field by Jones and Ransome (1993) who found a decrease by up to 1 kHz of f_rest_ in bats older than 10 years but otherwise in good body condition.

### Behavioural audiogram

Behavioural and neural audiograms describe relevant receiver properties in echolocating bats. In all previously studied flutter-detecting foragers, these audiograms are characterized by a sharply tuned minimum around f_ref_, which results in a maximal activation of the foveal areas of the hearing system (Rhinolophids: Neuweiler 1970; Schnitzler et al. 1971; Long and Schnitzler 1975; Schnitzler et al. 1976; Schuller 1980; Henson et al. 1985; Taniguchi 1985; *P. parnellii*: Grinnell 1970; Pollak et al. 1972; Suga et al. 1975; Henson et al. 1985; Hipposiderids: Grinnell and Hagiwara 1972; Schuller 1980; Neuweiler et al. 1984; for reviews, see Neuweiler et al. 1980; Schnitzler and Denzinger 2011). The behavioural auditory threshold of *R. paradoxolophus* measured with the Preyer reflex in this study strongly resembled the characteristics of these audiograms. We found a sharply tuned threshold minimum at 29.3 kHz, which is close to f_ref_, as estimated from the measured f_rest_ of 29.16 kHz. The maximum about 500–750 Hz just below the threshold minimum covers the frequency range of the emitted CF_2_ during flight, e.g., at flight speeds up to 3.8 m/s, the CF_2_ is 625 Hz below f_ref_. This maximum is characteristic for all DSC bats studied so far, and is thought to reduce vocal self-stimulation by emitted signals (Grinnell 1970; Neuweiler 1970; Suga et al. 1975). A high threshold around the first harmonic found in other rhinolophids and hipposiderids was also apparent in *R. paradoxolophus*, around 15 kHz. In between these two maxima there was another broader sensitive region, also reported in audiograms of *R. ferrumequinum*, *R. ferrumequinum nippon*, and *R. rouxi* (Neuweiler 1970; Neuweiler et al. 1971; Long and Schnitzler 1975; Taniguchi 1985; Kössl 1994). In *R. ferrumequinum*, this broadly expanded minimum is located around 60 kHz, and allocates to the terminal FM, which has bandwidths of 13–22 kHz (Long and Schnitzler 1975; Tian and Schnitzler 1997). In *R. paradoxolophus*, a broader sensitive region at 26.3 kHz, and 3 kHz below the audiogram minimum corresponded exactly to the terminal FM, which had a bandwidth of 2–4 kHz. Above the minimum close to f_ref_, the auditory threshold of *R. paradoxolophus* increased slightly by 3–5 dB SPL up to a frequency of 31.3 kHz, followed by a steep rise. In higher frequencies of 35–95 kHz, the thresholds remained high with 70 dB SPL on average (range from 51 to 99 dB SPL). We did not find another threshold lowering in the range of higher harmonics. The audiograms measured in other species did not include frequencies of these higher harmonics.

## Conclusions

Overall, the high precision with which *R. paradoxolophus* adjusted the echoes of targets from ahead at f_ref_, along with the sharp tuning of the behavioural audiogram to f_ref_, indicates a tight match between functional transmitter and receiver properties in the low frequency echolocation system of this species. This functional match is based on the morphologically tight link between foveal areas of hearing and vocalizing centres in the brain, and supports our current view on the function of the audio–vocal control system for DSC in flutter-detecting foragers (Schoeppler et al. 2018; 2022). The reference frequency (f_ref_) which is the controlled process variable of the audio–vocal control system in flutter-detecting foragers, is not a fixed frequency value but rather is determined by the activation state of the morphologically defined foveal area in the cochlea and in the connected higher foveal centres of the hearing and the vocal control system. DS compensating bats readjust the emission frequency (and with it f_ref_) if the cochleotopic input is changed, and the reported activation state of the foveal area differs from the set point state of the vocal control system. A change of the vibration properties of the fovea by changes in body temperature or by long term morphological changes, e.g., via aging, will lead to a concomitant change in f_ref_. The shift of f_ref_ in bat 1 by 700 Hz over 12 months suggests just such a change in the vibration properties of the cochlea.

## Supplementary Information

Below is the link to the electronic supplementary material.

Supplementary file1 (AVI 17302 KB)

Supplementary file2 (PDF 762 KB)
