# Supplementary material for: Transmitter and receiver of the low frequency horseshoe bat Rhinolophus paradoxolophus are functionally matched for fluttering target detection
Source: J Comp Physiol A Neuroethol Sens Neural Behav Physiol. 2022 Sep 22;209(1):191–202. doi: 10.1007/s00359-022-01571-0 (PMC9898408; doi:10.1007/s00359-022-01571-0)
Supplement: Supplementary file 2 — Supplementary file2 (PDF 762 KB) [file 359_2022_1571_MOESM2_ESM.pdf]

Supplementary information

Transmitter and receiver of the low frequency horseshoe bat *Rhinolophus paradoxolophus* are functionally matched for fluttering target detection

Journal of Comparative Physiology A

Diana Schoeppler[1*], Katrin Kost[1], Hans-Ulrich Schnitzler[1] and Annette Denzinger[1]

Authors affiliations:

[1] Animal Physiology, Institute for Neurobiology, University of Tübingen, Germany

*Corresponding author: diana.schoeppler@uni-tuebingen.de

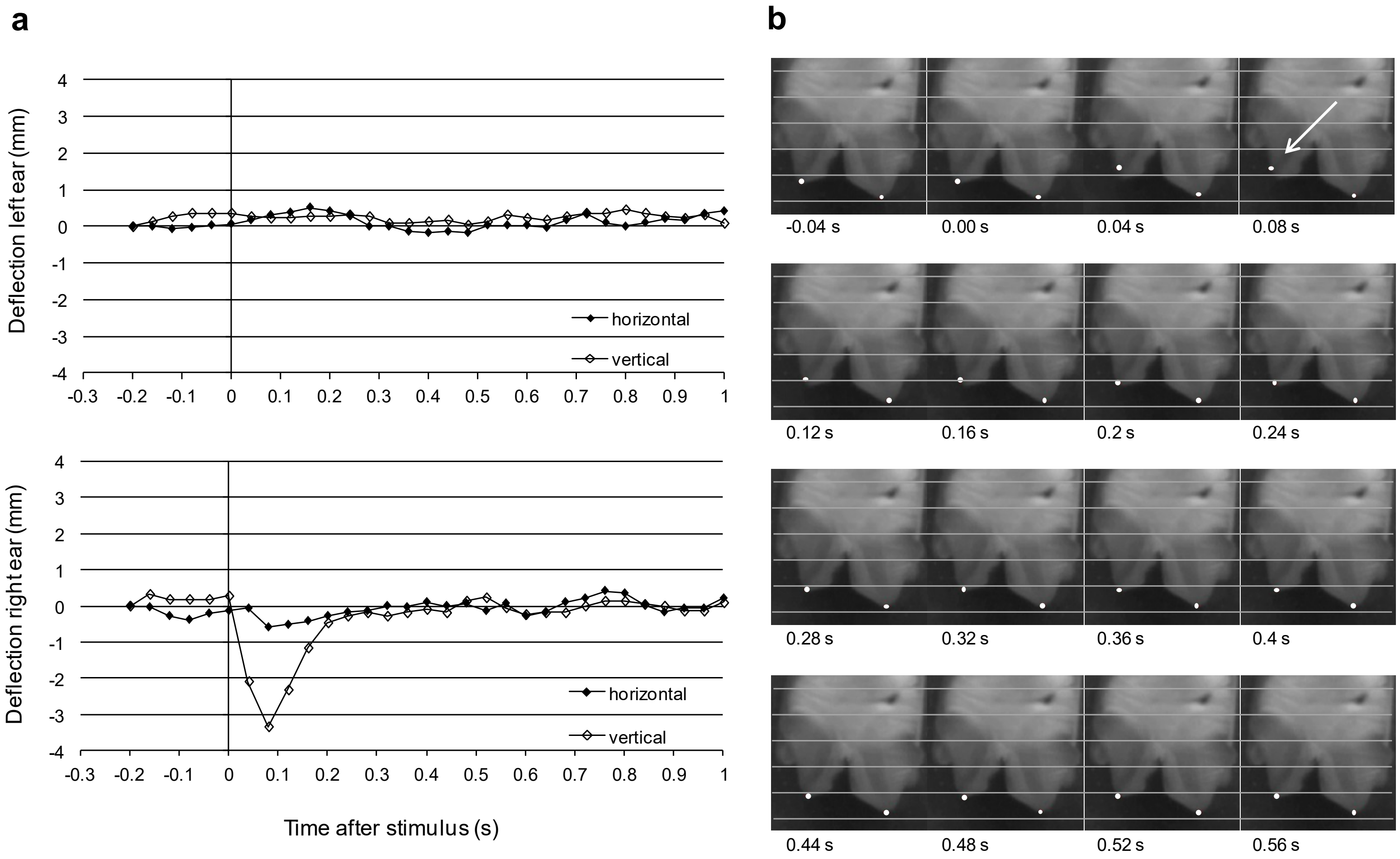

Fig. S1: Ear movement of *R. paradoxolophus* as reaction of a pure tone stimulus of 55 kHz at 70.5 dB SPL. The graphs display the horizontal and vertical deflection of the left and right pinna (a). The stimulus was presented at time 0. Frames of the video recording (b) show the reaction 40 ms before and 560 ms after the stimulus representation (shaded area in a). Tips of the pinnae are marked with a white dot. The arrow indicates the reaction.
